# Challenges and tackles in metabolic engineering for microbial production of carotenoids

**DOI:** 10.1186/s12934-019-1105-1

**Published:** 2019-03-18

**Authors:** Chonglong Wang, Shuli Zhao, Xixi Shao, Ji-Bin Park, Seong-Hee Jeong, Hyo-Jin Park, Won-Ju Kwak, Gongyuan Wei, Seon-Won Kim

**Affiliations:** 1 School of Biology and Basic Medical Sciences, Soochow University, 199 Renai Road, Suzhou, 215123 People’s Republic of China; 2 Division of Applied Life Science (BK21 Plus), PMBBRC, Gyeongsang National University, 501 Jinju-daero, Jinju, 52828 Republic of Korea

**Keywords:** Carotenoids, Synthetic biology, Systems biology, Membrane engineering

## Abstract

Naturally occurring carotenoids have been isolated and used as colorants, antioxidants, nutrients, etc. in many fields. There is an ever-growing demand for carotenoids production. To comfort this, microbial production of carotenoids is an attractive alternative to current extraction from natural sources. This review summarizes the biosynthetic pathway of carotenoids and progresses in metabolic engineering of various microorganisms for carotenoid production. The advances in synthetic pathway and systems biology lead to many versatile engineering tools available to manipulate microorganisms. In this context, challenges and possible directions are also discussed to provide an insight of microbial engineering for improved production of carotenoids in the future.

## Introduction

Carotenoids are naturally occurring products initially recognized as colorants owing to absorption of blue light and appearance of yellow to red colors. To date, plenty of these compounds (> 1100) have been found from many species of bacteria, eukaryotes, and some archaea [1]. They are built up from five-carbon (C_5_) isoprene units and rearranged to form long-chain carotenes or xanthophyll, which contain several conjugated double bonds. Besides as colorants, this characteristic chemical structure features their physiological functions as antioxidants, provitamin A nutrients, and UV protection agents. A medical potential has been also observed in prevention of chronic diseases and tumors [2]. With the roles in redox homeostasis and human health [3], they are widely used in industry as food ingredients, cosmetic bioactive substances, and medicinal compounds. C_40_ carotenoids are the major ones (> 95%), where lycopene, β-carotene zeaxanthin, lutein, β-cryptoxanthin are well known carotenoids in human diets [4]. Global market for carotenoids has reached $1.5 billion in 2017 and is expected to grow to $2.0 billion by 2022 [5]. There is limited production of carotenoids from natural resources [6]. Thus, microbial production of carotenoids is a prospective alternative way to meet an ever-growing demands [7, 8] because microbial production is easily scalable with increase of fermentation capacity and not affected by variations of season, climate, regions, etc. Moreover, microbial synthesis affords more bioactive products than chemical synthesis owing to region- and stereo-selective enzymes. In this review, we summarize current advances and the challenges in microbial engineering for carotenoid production.

## Biosynthetic pathways of carotenoids

**Fig. 1:**
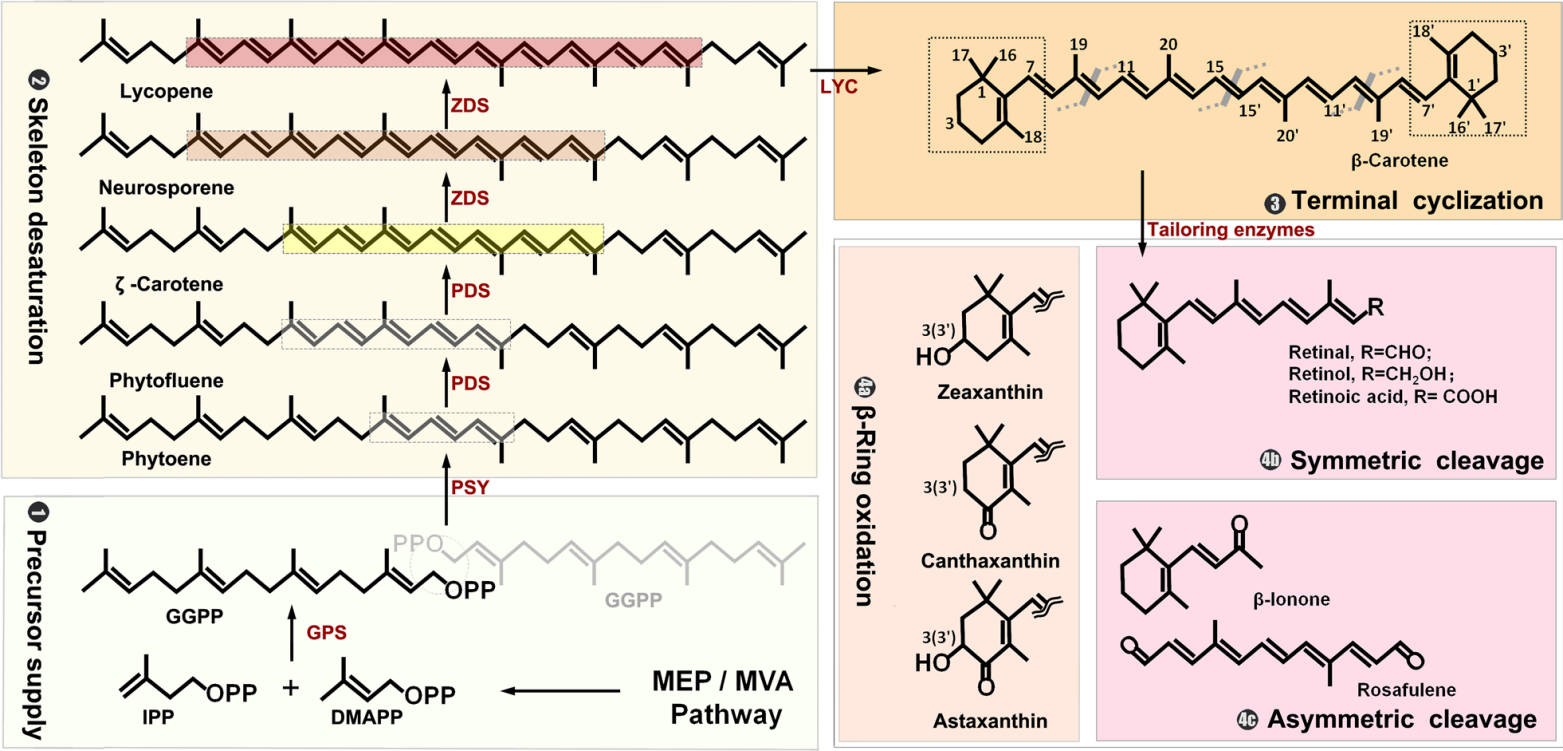
Scheme of carotenoids biosynthesis. Carotenoids synthesis can be divided into four stages: precursor supply, skeleton desaturation, terminal cyclization, and product tailoring. The conjugated double bands (c.d.b.) formed in desaturation stage are boxed with different color. The numbering of C_40_ carotenoids is exemplified by using β-carotene. The symmetric oxidations of β-ring generate zeaxanthin, canthaxanthin, and astaxanthin. The cleavage positions are presented on β-carotene, which leads to the symmetric products of retinoids and the asymmetric product of ionones. *GPS* GGPP synthase, *PSY* phytoene synthase, *PDS* phytoene desaturase, *ZDS* ζ-carotene desaturase, *LYC* lycopene cyclase

Biosynthesis of carotenoids can be divided into four steps: geranylgeranyl diphosphate (GGPP, C_20_) precursor supply, phytoene desaturation, lycopene cyclization, and carotene modifications (Fig. 1). Mevalonate (MVA) pathway in eukaryotes and some bacteria, and the 2-C-methyl-d-erythritol 4-phosphate (MEP) pathway in most bacteria and plant plastids are engaged in synthesis of isoprene units, dimethylally diphohsphate (DMAPP, C_5_) and isopentenyl diphosphates (IPP, C_5_). One DMAPP and three IPPs are condensed into one GGPP in a series of prenyltransferase reactions. Variation of the prenyltransferases of the condensation reactions can alternate the precursor to farnesyl diphosphate (FPP, C_15_) and geranylfarnesyl diphosphate (GFPP, C_25_), which serve as the precursors of minor C_30_ and C_50_ carotenoids. A linear colorless phytoene is initially generated from head-to-head condensation of two GGPPs by phytoene synthase (PSY), which serves as a gatekeeper of C_40_ carotenoid synthesis [9]. In general, phytoene undergoes a four-step desaturation by phytoene desaturase (PDS) and ξ-carotene desaturase (ZDS), and two complementary isomerizations by 15-*cis*-ζ-carotene isomerase (ZISO) and carotenoid isomerase (CRTISO), which results in the formation of a red-colored lycopene with the fully conjugated undecane chromophore [10]. In contrast, bacterial carotene desaturase, called CrtI, solely takes over these four-step desaturations [11]. The stepwise desaturation lead to intermediates with c.d.b. number from 3 to 11, which renders different colors when the c.d.b. number exceeds seven [12]. Different lycopene cyclases then catalyze to introduce β- or ε-ring at chain ends of lycopene to form α-, β-, γ- or δ-carotene according to a double bond position of the ionone ring [13, 14]. These carotene products present diversity in geometrical skeleton of carotenoids, which can be hydroxylated at the terminal ionone ring by hydroxylase and ketolases to form various xanthophylls. For example, the addition of hydroxyl groups to C-3(3′) position of β-carotene yields zeaxanthin, while 4(4′)-ketolation of β-carotene results in canthaxanthin product. The oxidative C-3(3′) and/or C-4(4′) positions of β-carotene can happen in many combinatorial orders to generate intermediates of astaxanthin [15]. There are also further modifications of carotenoids such as glycosylation (e.g., myxol 2′-fucoside) and acetylation (e.g., dinoxanthin) not discussed herein. These carotenoids can be oxidatively cleaved to generate apocarotenoids, including retinoids and non-retinoids by carotenoid cleavage oxygenase/dioxygenases or 9-*cis*-epoxycarotenoid dioxygenases (NCEDs) [16]. The most renowned retinal is derived from symmetric cleavage of β-carotene at central (15,15′-) double bond, while asymmetric cleavages at 9,10- and/or 9′,10′- double bonds generate β-ionone, and β-*apo*-10′-carotenal or rosafluene. It could give a great interest to mine more pathways for production of apocarotenoids from parent carotenoids such as β-carotene and lycopene.

## Microbial production of carotenoids using a native producer

Microbial production of carotenoids is a promising way for economic and mass production of natural-origin carotenoids (Table 1). Fermentation of heterothallic *Blakeslea trispora* and *Phycomyces blakesleeanus*, native carotenogenic (*crt*) fungi, is known as a representative way of microbial production of carotenoids in an industrial scale [17]. The culture has been optimized in culture media and various fermentation parameters [18]. Manipulation of oxygen transfer rate (OTR) at 20.5 mM/L/h leads to β-carotene production of 704.1 mg/L, because reactive oxygen species (ROS) are formed in the high OTR condition and stimulates carotenoid synthesis [19]. A waste cooking oil is used as a cheap carbon source to produce more than 2 g/L of carotenes [20]. Lycopene production from *B. trispora* is achieved by addition of lycopene cyclase inhibitors such as 2-methyl imidazole, where 256 mg/L of lycopene was produced by using a bubble column reactor [21]. Microbial carotenoids production using a native producer are thus focused on isolation of robust strains able to use low-cost substrates and development of competitive bioprocess [22].

**Table 1:** Representative engineering strategies for carotenoid production from microbial hosts

Host strains	Descriptions	Products and titers	Engineering strategies	References
*Blakeslea trispora*	Native producer of carotenoids,	β-Carotene 704.1 mg/L	Control of oxygen transfer rate	[19]
		Lycopene, 256 mg/L	Optimization of fermentation with lycopene cyclase inhibitor	[21]
*Escherichia coli*	Genetically tractable, non-native producer	Lycopene, 0.5 g/g DCW	Regulation of lycopene synthesis pathway expression	[28]
		β-Carotene, 2.1 g/L	Engineering MEP pathway for IPP and DMAPP supply and central pathway (TCA, PPP) for carbon flux	[31]
*Saccharomyces cerevisiae*	Genetically tractable, non-native producer	Lycopene, 56 mg/g DCW	Increase of acetyl-CoA pool and optimization of lycopene synthesis pathway via genome manipulation	[34]
		Astaxanthin, 218 mg/L	Genome evolution by ARTP	[36]
*Corynebacterium glutamicum*	Native producer of C_50_ carotenoid	β-Carotene, 7 mg/L	Deletion of *crtR* and integration of *crt* pathway genes	[40]
*Rhodobacter sphaeroides*	Phototroph with carotenogenic genes	Lycopene, 10 mg/g DCW	Replacement of *crtI*, augmentation of MEP pathway, and block of PPP pathway	[43]
*Xanthophyllomyces dendrorhous*	Astaxanthin producer	Zeaxanthin, 0.5 mg/g DCW	Mutagenesis of astaxanthin synthase and overexpression of β-carotene hydrolase	[47]
*Yarrowia lipolytica*	Genetically tractable, non-native producer	β-Carotene, 6.5 g/L	Optimization of promoter-gene pairs of heterologous *crt* pathway	[53]
		β-Carotene, 4 g/L	Iterative integration of multiple-copy pathway genes	[52]

## Metabolic engineering of microbes for carotenoid production

With advances in metabolic engineering and synthetic biology, many efforts have been conducted to produce carotenoids from genetically tractable microorganisms (e.g., *Escherichia coli* and *Saccharomyces cerevisiae*), which is well reviewed elsewhere [23, 24]. Biosynthesis pathway of lycopene or β-carotene has been often introduced and engineered in non-carotenogenic hosts owing to available genetic collections of the pathways. Engineering strategies include enhancement of IPP and DMAPP precursors supply by overexpression of rate-limiting enzyme of the MEP or MVA pathway, optimization of heterologous *crt* pathway, and modification of host chassis. Balanced augmentation of IspG and IspH in MEP pathway could eliminate accumulation of the pathway intermediates, and improve lycopene and β-carotene production [25]. MVA pathway possesses great potential for isoprenoids production [26], and heterologous expression of MVA pathway increases β-carotene production to 465 mg/L in an engineered *E*. *coli* [27]. Thanks to colorimetric traits of carotenoids, synthetic pathways of carotenoids are often adopted for validation of designing concepts in metabolic engineering and synthetic biology. Thus, it provides many novel strategies to optimize *crt* pathways [28, 29]. A new combinatorial multigene pathway assembly scheme is implemented with use of well-characterized genetic parts of lycopene synthesis, resulting in lycopene production of 448 mg/g DCW [28]. *E. coli* is rationally evolved to accommodate lycopene production by multiplex automated genome engineering (MAGE) in a short time [30], ATP and NADPH supplies for β-carotene production are improved by engineering central metabolic modules of carbon sources assimilation (EMP and PPP pathways), which allows 2.1 g/L of β-carotene production from the engineered *E. coli* in a fed-batch culture [31]. As robust carotenoids production depends on a stability of carotenogenic pathway plasmids, engineering of the plasmids stability based on *hok*/*sok* system yields a reproducible production of 385 mg/L astaxanthin from recombinant *E. coli* [32]. To achieve a high-level, genetically stable expression of heterologous genes and pathways, chemically inducible chromosomal evolution (CIChE) is successfully applied to optimize genes dosage of chromosomal-integrated lycopene pathway in *E. coli* [33]. *S. cerevisiae* is engineered to produce lycopene through combining host engineering to increase acetyl-CoA pool and pathway engineering to optimize genes expression, resulting in a 22-fold increase in lycopene production (55.6 mg/g DCW) as compared to its initial strain [34]. An increase in availability of NADPH by overexpression of *STB5* transcription factor yields 41.8 mg/L of lycopene in *S. cerevisiae* with the engineering efforts to reduce ergosterol synthesis and to enhance MVA pathway [35]. A combined approach of heterologous carotenoids module engineering and mutagenesis by atmospheric and room temperature plasma (ARTP) could make *S. cerevisiae* produce 218 mg/L of astaxanthin [36].

## Development of microbial hosts for carotenoid production

With expansion of available synthetic biology tools various microorganisms are manipulated to produce carotenoids. *Corynebacterium glutamicum*, a native producer of C_50_ decaprenoxanthin, is well known for amino acid production as well as vigorous sugar utilization with less carbon catabolite repression [37]. A deletion of *crtR* in *C. glutamicum* results in derepression of *crt* operon and a several-fold increase in lycopene, β-carotene and decaprenoxanthin production [38]. Carotenoids production is also improved by overexpression of σ-factor (*sigA*) in *C. glutamicum* [39]. Simultaneous production of l-lysine, 1.5 g/L and β-carotene, 7 mg/L using xylose as alternative feedstock was obtained from *C. glutamicum* with a series of integrations of *crt* pathway and lysine pathway as well as deletion of *crtR* [40]. Purple bacterium *Rhodobacter sphaeroides* is a facultative anaerobic phototroph with a set of c*rt* genes for synthesis of spheroidene and spheroidenone [41]. *Rb. sphaeroides* has highly-invaginated membrane structure which would favor carotenoid deposition [42]. It was engineered to produce 10.32 mg/g DCW of lycopene by replacement of endogenous neurosporene hydroxylase (CrtC) with heterologous phytoene desaturase (CrtI) along with augmentation of MEP pathway and block of carbon flux to pentose phosphate pathway (PPP) [43]. Diploid *Xanthophyllomyces dendrorhous* is capable of astaxanthin synthesis. Overexpression of rate-limiting GGPP synthase by promoter engineering has improved astaxanthin content by 1.7 folds [44]. Deletions of diploid CYP61 genes encoding sterol desaturase could relieve feedback inhibition of ergosterol to MVA pathway, and promote astaxanthin production by 1.4 folds [45]. A mutagenic treatment generated its variants accumulating β-carotene [46], which was engineered for zeaxanthin production, 0.5 mg/g DCW by introduction of β-carotene hydroxylase [47]. *Yarrowia lipolytica*, an oleaginous yeast is an industrial organism for cost-effective production of compounds derived from acetyl-CoA [48]. There are many genetic tools developed to engineer *Y. lipolytica* over decades [49]. It is thus regarded as a promising host for production of carotenoids derived from acetyl-CoA via MVA pathway. A heterologous lycopene pathway was introduced in *Y. lipolytica* engineered to increase the size of lipid bodies by deletion of peroxisomal β-oxidation pathway, which favored lycopene deposits in the lipid bodies and increased the production [50]. Overexpression of MVA pathway and alleviation of auxotrophy in *Y. lipolytica* PO1f strain allow 21.1 mg/g DCW of lycopene production [51]. An efficient β-carotene pathway was generated by using strong promoters and multiple gene copies for the synthesis pathway, which brought 4 g/L of β-carotene production under optimized fed-batch culture [52]. A combinatorial approach based on Golden Gate assembly was implemented to optimize promoter-gene pairs of heterologous *crt* pathway, by which a best strain yielded 90 mg/g DCW of β-carotene with a titer of 6.5 g/L in fed-batch culture [53]. *Y. lipolytica* thus shows a great potential as a competitive host strain for production of carotenoids.

## Challenges and outlook in microbial production of carotenoids

**Fig. 2:**
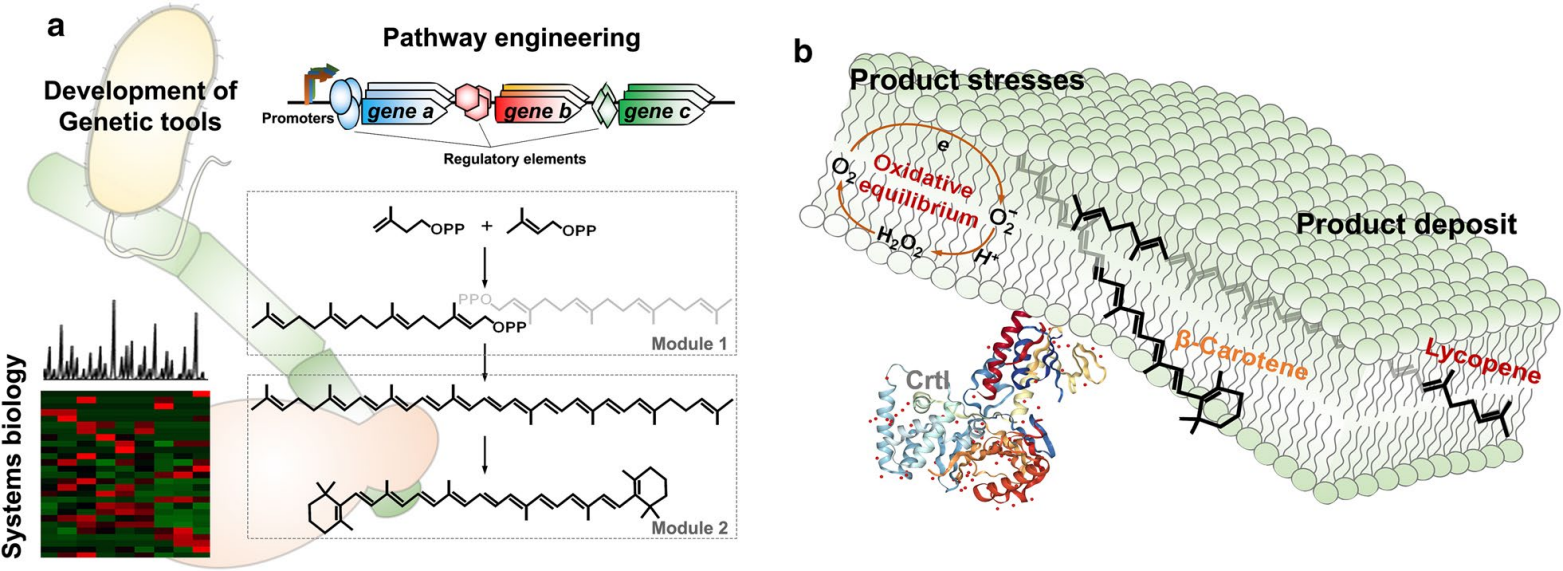
Possible engineering directions for microbial production of carotenoids. Many synthetic biology tools are developed and implemented to pathway engineering in many microbial hosts to increase carotenoid synthesis. Rational manipulation of synthetic pathway will rely heavily on systems biology to understand the cross-talk between carotenoid synthesis pathway and host metabolic network (**a**). Carotenoids and *crt* enzymes are associated with membrane. Membrane structural integrity and dynamics are requisite of cell survival, which restricts cellular accumulation of carotenoids (**b**). Representative structure of CrtI protein (PDB: 4DGK) is visualized by using NGL viewer

Metabolic engineering and synthetic biology have demonstrated many great successes in microbial production of carotenoids as aforementioned. Engineering efforts for the successes include overexpression of rate-limiting genes, blocking of competing pathways draining precursors, products or cofactors, and removal of endogenous regulatory loops. The engineering strategies are mostly employed for production of lycopene and β-carotene, because their synthesis pathways are well known and characterized to benefit proof-of-principle demonstrations in synthetic biology (Fig. 2a). As there are many industrially interesting carotenoids such as xanthophylls and apocarotenoids, the engineering efforts are required to address production of various carotenoids beyond lycopene and β-carotene, and their derivatives. Retinoids, symmetric cleavage products of β-carotene, are produced to 136 mg/L with introduction of β-carotene 15,15′-monooxygenase (BCMO) in *E*. *coli* engineered to produce β-carotene [54]. The retinoids composition can be modulated by overexpression of promiscuous endogenous oxidoreductase YbbO, reducing retinal to retinol [55]. α- and β-Ionone are asymmetric cleavage products of carotenoids. They are produced by employment of carotenoid cleavage dioxygenase (CCD) in an engineered *E*. *coli* producing ε- or β-carotene, where ~ 30 mg/L and ~ 500 mg/L of ionones were produced in flask and bioreactor, respectively [56]. Colorless phytoene and phytofluene, carotenoids precursors to lycopene, receive attentions in their nutritional aspects [12, 57]. Phytoene production can be attained by disrupting its desaturation step and yield of 7.5 mg/g DCW is reported in *X. dendrorhous* [58]. Rare C_50_ carotenoids have longer c.d.b. and superior antioxidative properties than C_40_ carotenoids, whose synthesis is observed from few microorganisms in a low titer [9, 59]. Production of C_50_ carotenoids is addressed by directed evolution of key *crt* enzymes determining carbon chain backbone lengths in C_40_ carotenoids synthesis pathway [60, 61]. A new perspective in microbial production of carotenoids could be expansion of catalog of carotenoids from usual carotenes to xanthophylls, apocarotenoids and rare carotenoids.

Most engineering strategies mainly focus on stoichiometric modulation of pathway components or enhancement of metabolic flux by pushing and pulling, which are also applied successfully to carotenoids production. However, a distinctive engineering strategy is required for carotenoids production beside on the general strategies because some of enzymes in carotenogenic pathways and their substrates are present in membrane regions (Fig. 2b). Lycopene is a desaturated product by lipophilic phytoene desaturases, plastidal PDS/ZDS or bacterial CrtI [10, 11]. Both the plant and bacterial enzymes are translocated to membrane after translation, and CrtI activity depends on a concerted process of flavin-binding and membrane association [62, 63]. Thus, engineering of membrane localization of *crt* pathway components can favor them to access their hydrophobic substrates and redox partners located in the membrane. β-Carotene ketolase and hydroxylase are engineered to co-localize in membrane through fusion with glycerol channel protein (GlpF), which increases astaxanthin production by 2.2 folds [64]. Carotenoids production would be restricted at a certain extremity level by limited storage space and product toxicity, caused by carotenoids deposition in cytosolic membrane. As carotenoids are deposited in cellular membranes or neutral lipid droplets, a maximized microbial production of carotenoids could be obtained by expansion of cellular membranes or augmentation of neutral lipid droplets formation.

Heterologous carotenoids synthesis pathways have been knocked into chromosomes of host microorganisms by genome engineering tools such as Cas9-CRISPR for stable maintenance of the heterologous pathways with no use of antibiotic selection pressure [65, 66], resulting in a stable production of β-carotene, 2.0 g/L in *E. coli* [67]. Cas9-CRISPR tool has been developed in many species such as *S. cerevisiae* and *Y*. *lipolytica* [68–70]. Rewiring of host metabolism in a genome level could be attained conveniently with CRISPR tools enabling knock-down or knock-out of chromosomal genes as well [71]. Glycolytic pathway generates acetyl-CoA with a theoretical carbon yield of 66.7% owing to pyruvate decarboxylation. Maximum carbon yield of MVA pathway could not be higher than 55.6% because of carbon loss at decarboxylation of mevalonate diphosphate as well as the loss of the glycolytic pathway. Rerouting carbon flux using PPP can minimize carbon loss and improve yield of isoprenoids [72]. Recent genetic tools including Cas9-CRISPR help metabolic engineers rewire metabolisms of various hosts at ease. Systems biology promises a comprehensive understanding of whole metabolic process with collective measuring of various cellular components such as entities of RNAs, proteins, and metabolites [73]. The multi-level Omics data are often integrated with computational approaches together to discriminate varying production phenotype in a holistic manner [74, 75]. It would be of the most interest to know the stresses from a featured *crt* pathway and carotenoid products as well as host response to them, which could in turn guide optimization of host engineering.

Overall, carotenoids show many benefits in health, nutrition, and better well-being. Microbial production of carotenoids is the alternative to us to meet the ever-growing demand of these valuable compounds. It is envisaged expansion of microbial carotenoids catalog by mining pathways which have not yet been identified, and manipulation of microbial hosts based on understanding of how cells accommodate carotenoids (Fig. 2). Efforts towards the challenging issues will be an important basis for industrial production of carotenoids. With emerging synthetic biology and systems biology tools, more progresses will be made to develop microbial cell factories for mass production of carotenoids in the future.
